# ICTV Virus Taxonomy Profile: *Aoguangviridae* 2023

**DOI:** 10.1099/jgv.0.001922

**Published:** 2023-11-27

**Authors:** Yifan Zhou, Yongjie Wang, Mart Krupovic

**Affiliations:** ^1^​ Institut Pasteur, Université Paris Cité, Archaeal Virology Unit, Paris 75015, France; ^2^​ Sorbonne Université, Collège Doctoral, Paris 75005, France; ^3^​ College of Food Science and Technology, Shanghai Ocean University, Shanghai, PR China

**Keywords:** ICTV Report, virus taxonomy, magroviruses, archaeal *Caudoviricetes*, marine archaea, *Magrovirales*

## Abstract

The family *Aoguangviridae* includes dsDNA viruses that have been associated with marine archaea. Currently, members of this virus family are known through metagenomics. Virions are predicted to consist of an icosahedral capsid and a helical tail, characteristic of members in the class *Caudoviricetes*. Aoguangviruses have some of the largest genomes among archaeal viruses and possess most of the components of the DNA replication machinery as well as auxiliary functions. The family *Aoguangviridae* includes the species *Aobingvirus yangshanense*. Many unclassified relatives of this virus group, referred to as ‘magroviruses’, have been discovered by metagenomics in globally distributed marine samples. This is a summary of the International Committee on Taxonomy of Viruses (ICTV) Report on the family *Aoguangviridae*, which is available at ictv.global/report/aoguangviridae.

## Virion

Members of the family *Aoguangviridae* have been discovered through metagenomics and have yet to be cultured [1–4] (Table 1). However, based on the conservation of the structural module typical of bacterial and archaeal viruses of the class *Caudoviricetes* [5] and including the HK97-fold major capsid protein, capsid maturation protease, large subunit of the terminase, portal, major tail protein, baseplate, etc., virions are predicted to consist of an icosahedral capsid and a helical tail. The lack of the gene encoding a tail sheath protein suggests that the tails of aoguangvirids are not contractile.

**Table 1.** Characteristics of members of the family *Aoguangviridae*

Example	Poseidoniales virus YSH_150918 (ON649702), species *Aobingvirus yangshanense*, genus *Aobingvirus*
Virion	Predicted to have virions characteristic of members of the class *Caudoviricetes*, with icosahedral capsids and helical tails
Genome	Linear, dsDNA genome of 92.2 kbp
Replication	Virus encoded DNA replisome, including family B DNA polymerase
Translation	Unknown
Host range	Marine archaea
Taxonomy	Realm *Duplodnaviria*, kingdom *Heunggongvirae*, phylum *Uroviricota*, class *Caudoviricetes*, order *Magrovirales*: the family includes the genus *Aobingvirus* and the species *Aobingvirus yangshanense*

## Genome

**Fig. 1.**
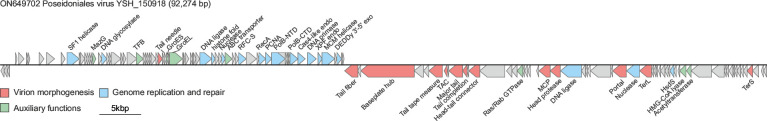
Genome organisation of virus YSH_150918. Arrows depict open reading frames indicating the direction of transcription. Abbreviations: SF1, superfamily 1; TFB, transcription factor B; RFC-S, replication factor C, small subunit; PCNA, proliferating cell nuclear antigen/DNA polymerase sliding clamp; PolB-NTD/CTD, family B DNA polymerase, N-/C-terminal domain; endo, endonuclease; MCM, minichromosome maintenance; exo, exonuclease; TAC, tail assembly chaperone; MCP, major capsid protein; TerS/TerL, small/large subunit of the terminase; HsdS, type I restriction modification system specificity (S) subunit; HMG-CoA, β-Hydroxy β-methylglutaryl-CoA.

The genome of aoguangvirids is double-stranded DNA of 90–100 kbp, one of the largest described for archaeal viruses. The 92.2 kbp genome of virus YSH_150918 has been assembled as a circular molecule, suggesting terminal redundancy and/or circular permutation. The genome has a G+C content of 31.5 % and encodes 129 predicted proteins [3]. The genes encoding functions related to genome replication and virion morphogenesis are organized into two clusters which are predicted to be transcribed in opposite directions (Fig. 1).

## Replication

No member of the family *Aoguangviridae* has yet been cultured and so insights into their biology can be only gleaned from computational analysis of the virus genome. Aoguangvirids do not encode an identifiable integrase or transposase and are likely to lead a lytic lifestyle. A characteristic feature of aoguangvirids, also observed in other, unclassified members of the order *Magrovirales*, is the extensive set of genes required for genome replication and repair [1–3]. In particular, virus YSH_150918 encodes a family B DNA polymerase, archaeo-eukaryotic DNA primase, DNA polymerase sliding clamp, clamp loader, replicative minichromosome maintenance helicase, ATP-dependent DNA ligase, and other proteins [3]. Thus, it is likely that aoguangvirids replicate their genome with little, if any, requirement for host replication factors. Besides the genes required for genome replication and repair, as well as virion morphogenesis, aoguangvirids encode auxiliary functions implicated in protein folding (e.g., chaperonin GroEL and cochaperonin GroES) and modulation of the host metabolism (e.g., β-Hydroxy β-methylglutaryl-CoA lyase) (Fig. 1).

## Taxonomy

**Fig. 2.**
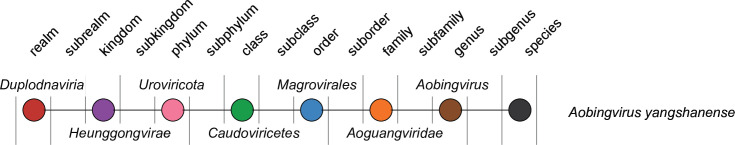
Relationships of the taxa connected to the family *Aoguangviridae*.

Current taxonomy: ictv.global/taxonomy. The family *Aoguangviridae* includes the genus *Aobingvirus* and the species *Aobingvirus yangshanense* (Fig. 2). Viruses in the family *Aoguangviridae* are most closely related to unclassified archaeal tailed viruses associated with marine archaea of the order *Poseidoniales *, formerly known as Marine Group II Euryarchaeota [1, 2], and more distantly related to viruses of the family *Druskaviridae* infecting hyperhalophilic archaea [6].

## Resources

Full ICTV Report on the family *Aoguangviridae*: ictv.global/report/aoguangviridae.
